# What do dental college clinicians know about oral cancer and its risk factors? An assessment among final year students, interns and faculty members in saudi arabia

**DOI:** 10.4317/jced.55168

**Published:** 2018-09-01

**Authors:** Mohammed Jafer, Rik Crutzen, Alhassen Jafer, Bart van den Borne

**Affiliations:** 1 Researcher (PhD. Candidate). Department of Preventive Dental Science, College of Dentistry, Jazan University, Saudi Arabia; 2 Associate professor. Department of Health Promotion, Maastricht University/CAPHRI, The Netherlands; 3 Dentist. Division of Dentistry, Ministry of Health, Jazan, Saudi Arabia; 4 Emeritus professor. Department of Health Promotion, Maastricht University/CAPHRI, The Netherlands

## Abstract

**Background:**

The ability of health care professionals to perform oral cancer examination depends partly on their knowledge of the disease and its risk factors. This study aimed to assess and compare the knowledge of final year students, interns and faculty members regarding oral cancer and its risk factors.

**Material and Methods:**

A 35-item questionnaire about knowledge of oral cancer and its risk factors was distributed among participants from Jazan University’s Dental School. A minimum score of 18 was the cut-off point for an acceptable total score of oral cancer knowledge [OCTS]. Descriptive statistics described the relations between demographics variables and knowledge levels of participants. The differences between OCTS, diagnostic-clinical examination knowledge [DCK] and supportive knowledge [SK] and sex and occupation were analyzed using independent t-test and ANOVAs respectively.

**Results:**

A total of 72 students, 68 interns and 88 faculty members completed the questionnaires (N = 228); with an average age of 23.8 ± 0.9 years, 25.1 ± 1.5 years and 40.6 ± 9.1 years with 55.1% males. OCTS was 20.2 ± 3.6 out of 35. No significant difference between OCTS and participants’ sex was found (t (203) = 1.342, *p* = .181, 95% CI for difference -.302 ــ 1.589). No significant differences in OCTS between students, interns and faculty members (F (2, 225) = 2.116, *p* = .123). A significant difference in SK between final year students, interns, and faculty members was founded (F (2, 194) = 5.62, *p* = .004).

**Conclusions:**

Knowledge of oral cancer and its risk factors among Jazan Dental School’s final year students, interns and faculty members is acceptable. However, due to the high rate of oral cancer in Jazan, emphasizing knowledge of oral cancer and its risk factors in the curriculum of Jazan Dental School is required.

** Key words:**Oral cancer, risk factors, knowledge, dental education, curriculum, dental students, dentists.

## Introduction

Oral cancer (OC) is a debilitating disease with serious impacts on individuals, families and societies. Although cancer treatment opportunities are advancing, there is also an increase in the incidence of OC (1) with 5-year survival rates remaining low; 50% (2). According to WHO, OC is the eighth most common cancer worldwide (3). The rate of OC varies among countries with high incidence rates of OC being reported in developing countries (3). Of all Gulf countries, Saudi Arabia exhibits the highest prevalence of OC cases (4). According to the latest published data from the Saudi Cancer Registry in 2014, cancers related to oral cavity are the highest registered cancer cases from the Jazan region of Saudi Arabia (5). The causes of this still unknown.

OC is usually asymptomatic and frequently diagnosed at late stage of its development (6,7). However, early detection of OC leads to better survival rates for individuals. Previous studies have confirmed reduced survival rates for higher stages of the disease at diagnosis (8,9). Furthermore, the delay in detection of OC was found to be associated with poor prognosis of such disease (9,10). Health care providers (HCPs) knowledge of OC and its risk factors is an important element regarding OC examination (11), and was found to be strongly associated with performing OC examination (12,13). Moreover, it was found that around one fourth of the OC patients had improper diagnosis of their conditions by their health care provider (14). The delay in OC diagnosis and the misdiagnosis of OC lead to poor prognosis (15,16), which was found to be associated with lack of knowledge regarding OC among health care providers (17). Indeed, HCPs knowledge of OC and its risk factors is essential for their behavior related to OC practice (18,19).

Additionally, dentists should be the first line of diagnosing OC (20) and therefore they have a duty to have good knowledge of OC and its risk factors, particularly important if they are working in area with high rates of OC incidence and prevalence. Thus, the objective of the present study was to investigate the knowledge level of final year student and dentists working at both, male and female, branches of the School of Dentistry; Jazan University, regarding OC and its risk factors. The present study also serves to investigate the differences in knowledge level of OC and its risk factors among different sex and different occupations of the participants. In addition, the same analyses were carried out for Saudi participants, whom are the majority of final year students and interns and whom will mostly work in the Jazan region of Saudi after their graduation. To our knowledge, this is the first study to assess the knowledge of health care professionals in the field of oral cancer in the Jazan region, Saudi Arabia.

## Material and Methods

-Participants and procedure

A descriptive cross-sectional study; using a convenience sampling technique, was conducted among all final year students, all interns and all faculty members, of the male and female branches of the dental school at Jazan University (JDS). Participants were informed and invited to participate in the study by the JDS administration office. To avoid any potential bias, all final year students, interns and faculty members’ participants, of both JDS male and female branches, who agreed to participate in the present study, were invited to the JDS main auditorium at the same time, which was divided into male and female sections as requested by JDS. All participants signed the consent form and questionnaires were distributed among the participants who filled the questionnaire on January 18, 2017 at 12:00 noon. All questionnaires were administrated by the main investigator (MI) and three assistants of compatible sex for each section from the school administration employees. All distributed questionnaires were collected after approximately 60 minutes. Participation was voluntary with no incentives for participation.

-Measurement and pretesting

A knowledge scale measuring knowledge of OC and its risk factors was developed by the MI based on current literature regarding OC and its risk factor, in addition to specific OC issues (high prevalence and its gender distribution) in Jazan region, from related published data and the Saudi Cancer Registry (5,21). The knowledge scale focused on investigating diagnostic-clinical examination knowledge (DCK) and supportive knowledge (SK) regarding OC, such as epidemiology, etiology, risk factors, prevention, management and prognosis. Subsequently, the knowledge scale was evaluated by three senior faculty members specialized in oral medicine, oral pathology and maxillofacial surgery from JDS, in terms of completeness, accuracy, and relevance of information included in the items. The final knowledge scale consisted of 35 items to investigate the level of knowledge among participants; with 13 DCK and 22 SK. The main outcome was established as the total score of oral cancer knowledge [OCTS], which is a sum of all correct answers; each correct answer was counted as one point. Due to the fact that participants were of different knowledge levels, experience and specialization, and due to some questions being of advanced level (i.e., Q4, Q 5, Q 7, Q 22/4) or requiring numeracy skills (i.e., Q 1, Q 11, Q 22/3), a minimum score of 18 was decided by the MI and the evaluators to be a cut-off point for acceptable OCTS, which is just above half of the total OCTS. The final questionnaire also included additional questions measuring age, sex, occupation of participants (final year student, intern, faculty member) and nationality. Subsequently, a pretest with fifteen participants from the targeted sample was conducted to identify problems with wording, understanding and interpretation of items, burden and duration and organization and item sequence of the questionnaire. Accordingly, questions number Q 1, Q 13, Q 20 and Q 23 were rephrased to more familiarized wording and all questions with true/false answer options were bloated for more visibility. Subsequently, the questionnaire was distributed among all participants to collect data on their knowledge of OC and its risk factors. The full questionnaire is available at <https://osf.io/umk4h/>.

-Data Analysis 

An overall picture of knowledge level regarding the OC among final year students, interns, and faculty members at the School of Dentistry, was obtained by means of descriptive statistics. Furthermore, due to the fact that there are two branches of JDS, male and female, which could lead to different OC educational exposure as lectures and clinical exposure and experience could vary for more than one factor, for example, different faculty members, clinical instructors and patients, we used independent t-tests to investigate the relation between each of OCTS, DCK or SK, and sex. This was also relevant because most of the questions in the DCK are of basic diagnostic and clinical knowledge and most of the advanced and epidemiological questions are in the SK. Due to differences in experience levels, ANOVA was used to investigate the relation between each of OCTS, DCK or SK, and occupation. Furthermore, because we were also interested in investigating the level of OC knowledge among the future dentists in Jazan region, the same tests were done among the sub-sample of Saudi participants only, because almost all students and interns were of Saudi nationality. The level of significance for the analytical tests was set at an alpha of .05 and we report 95% confidence intervals.

-Ethical Approval

Ethical approval was obtained from Jazan University before conducting the present study; Registry no. [CDREC-06], dated 21 December 2016.

## Results

All final year students, interns and faculty members from the dental college of Jazan University were approached for the present study; the total number was 263 members. Out of the 263 individuals whom were approached, 237 participated (90% response rate); 9 did not complete the questionnaire and were excluded from the analysis. The sample consisted of 72 out of 80 students (90%), 68 out of 70 interns (97%) and 88 out of 113 faculty members (78%). Of the sample, 55.1% was male and 44.9% was female with average students age of 23.8 ± 0.9 years (range of 22-26), interns age 25.1 ± 1.5 years (range of 24-32) and faculty members age 40.6 ± 9.1 years (range of 25-65). The participants were from five nationalities, as shown in  Table 1. The average total score of the participant knowledge regarding oral cancer, OCTS, in the present study was 20.2 ± 3.6 out of 35.

**Table 1:**
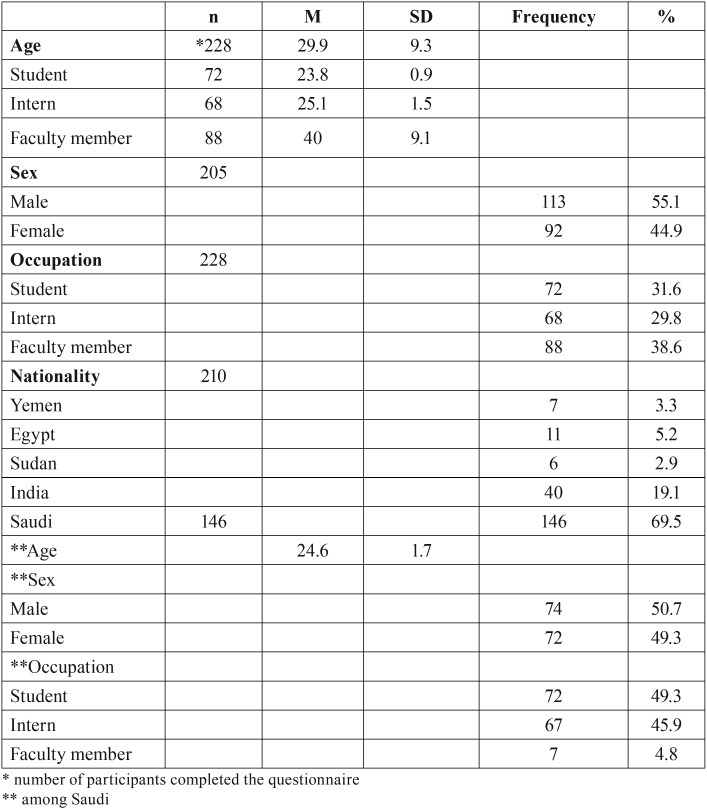
Demographics of the sample.

No significant relation between OCTS and the sex (male M = 20.5, SD = 3.53 and female M = 19.9, SD = 3.32) of the participants were found in the sample; there was no difference in sex (t (203) = 1.342, *p* = .181, 95% CI for difference -.302 ــ 1.589, d = 0.175). Moreover, there was no significant difference in occupation in terms of being students, interns or faculty members (F (2, 225) = 2.116, *p* = .123, ηp2 = 0.18; see  Table 2).

**Table 2:**
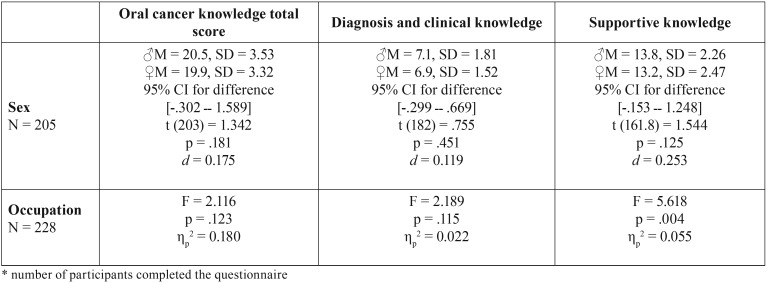
Individual statistical tests (N = 228*).

Furthermore, out of 13 questions investigating diagnosis and clinical knowledge regarding OC, the score of the sample ranged between 2 and 12 correct answer with mean of 7 ± 1.7. And out of 22 questions investigating other supportive knowledge regarding OC, the score ranged between 8 and 20 correct answers with a mean of 13.6 ± 2.4. Among participants of all different occupations, items Q15, Q16-2, Q19, Q21-1, Q21-2, Q21-3, Q22-3 and Q23 have the highest correct answer, and Q1, Q4, Q5, Q11, Q14, Q18-1, Q18-2 and Q20 have the lowest correct answer <https://osf.io/umk4h/>. Bivariate analysis did not reveal any significant differences for sex and occupation in the present study with regards to diagnosis and clinical knowledge (DCK). However, supportive knowledge (SK) was found to have a significant difference in the average scores for occupation (final year students’ M =13.5, SD=2.31, interns’ M = 12.9, SD = 2.39 and faculty members’ M = 14.3, SD = 2.19; F (2, 194) = 5.62, p .004, ηp2 = 0.055; see  Table 2).

Saudi’s accounted for 64% of the individual in the present study, with an average age of 24.6 ± 1.7 years. The male and female representation of the Saudis was almost equal [50.7% and 49.3% respectively]. Among them, final year students represented 49.3%, interns 45.9% and faculty members only 4.8%. There was no significant difference in OCTS average between Saudi male and female participants (male M = 20.1, SD = 2.81 and female M = 19.7, SD = 3.17; t (140.9) = 1.736, *p* = .406, 95% CI for difference -.567 ــ 1.394, d = 0. 134), nor were there any significant differences in DCK or SK. Moreover, within Saudis, there were no significant differences in OCTS average, diagnosis and clinical knowledge or supportive knowledge regarding OC with the different occupations ( Table 3). Furthermore, among Saudi participants, 95% answered question number 23 correctly, which was regarding risk factors of OC in region of Jazan. However, only 28% of them answered question 22.4 correctly, which was regarding risk factors of OC among females in the region of Jazan.

**Table 3:**
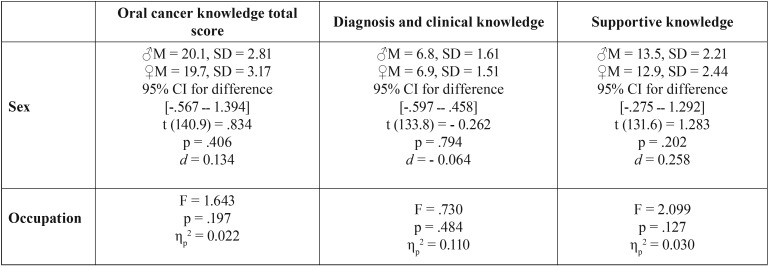
Individual statistical tests among Saudi (N = 146).

## Discussion

In general, most of dental treatments in dental school clinics are provided by their students and trainees, supervised by faculty. Similarly, in JDS, most of the dental treatments provided in its clinics are by the final year students and interns. Additionally, they are the first to examine and diagnose the majority of the JDS patients. Therefore, and because of the high prevalence of OC in the region, they need to have adequate knowledge of OC and its related risk factors and be competent in examining and assessing such disease. In the present study, the average knowledge of OC and its risk factors among last year students, interns and faculty members is at a moderate level; 20.2 ± 3.6, with no significant difference between them. The findings of the present study are in line with findings reported in the literature in regard to dental-students’ and dentists’ knowledge of diagnostics, clinical aspects, risk factors and prevention of OC (22-24).

Nevertheless, the majority of the participants (students, interns, faculty) had a high level of knowledge about how to preform OC examination but a low level of knowledge regarding the sites and clinical manifestation of the disease as well as it epidemiology. This is an important finding to be taking in consideration in the dentists’ education and clinical training, as it will affect their proper examination of OC. Therefore, JDS dental-students and dentists’ should have the essential knowledge of OC examination (12,13,25). However, besides this knowledge, dental-students and dentists will require clinical practice and experience to perform OC examination (26). The majority of the faculty members at JDS are from countries with similar OC burden, such as India, Yemen and Sudan (1). Those faculty members with their backgrounds and experiences could provide the best support to JDS towards training competent dentists in the field of OC and its related risk factors.

Although not significant, our study suggests that males had slightly better knowledge of OC (OCTS, DCK and SK) compared to females. These slight differences could be attributed to a higher number of male faculty members who were specialized in disciplines that incorporated OC advanced training. Similarly, among all Saudi participants, males had slightly better knowledge of OC (OCTS and SK) compare to females. These slight differences could also be due to the higher number of faculty members who received OC advanced training.

Additionally, due to the fact that most of the dentists in area of Jazan are from JDS, further investigation was carried to assess the level of knowledge related to OC and its local risk factors. Question 22.4, which was missed by the majority of Saudi participants, assessed the knowledge of participants regarding Jazan region OC special issue, where females have a slight higher rate of OC than males (5,21), which contradict with the international distribution (1). This could indicate that incorporate of OC and its local risk factors in the JDS curriculum is essential, as this different from OC international gender distribution.

The study carried some limitations as it was administrated in the middle of the academic year and knowledge of participants could be altered in different times of the academic year. Almost all final year students and interns have been taught by the same faculty members who participated in the present study. Some questions posed to the sample groups investigate knowledge that may not be required by final year students, interns and unspecialized dentists. However, the present study included 87% of the clinicians who are responsible for examining and educating patients in JDS clinics. The questionnaire was developed based on current knowledge and practice guidelines related to OC and its risk factors and were validated by three JDS faculty members who are competent in the field.

## Conclusions

Even though, JDS final year students, interns and faculty members have moderate levels of knowledge regarding OC and its risk factors, in the curriculum and clinical training of the dental school more emphasis should be put on increasing diagnostic-clinical and supportive knowledge and skills with respect to oral cancer. In the curriculum special attention should be paid to local risk factors and special needs in relation to oral cancer. Also, investigations of factors related to oral cancer and its high prevalence in Jazan and opportunities for primary prevention are highly recommended.
